# Changing Trends in Hospital Admissions for Pulmonary Embolism in Spain from 2001 to 2018

**DOI:** 10.3390/jcm9103221

**Published:** 2020-10-07

**Authors:** Javier de Miguel-Diez, Romana Albaladejo-Vicente, Ana Lopez-de-Andres, Valentín Hernández-Barrera, David Jiménez, Manuel Monreal, David Carabantes-Alarcon, José Javier Zamorano-Leon, Rodrigo Jimenez-Garcia

**Affiliations:** 1 Respiratory Department, Hospital General Universitario Gregorio Marañón, Instituto de Investigación Sanitaria Gregorio Marañón (IiSGM), Facultad de Medicina, Universidad Complutense de Madrid, CIBER de Enfermedades Respiratorias (CIBERES), 28040 Madrid, Spain; javier.miguel@salud.madrid.org; 2 Department of Public Health & Maternal and Child Health, Faculty of Medicine, Universidad Complutense de Madrid, 28040 Madrid, Spain; dcaraban@ucm.es (D.C.-A.); josejzam@ucm.es (J.J.Z.-L.); rodrijim@ucm.es (R.J.-G.); 3 Preventive Medicine and Public Health Teaching and Research Unit, Health Sciences Faculty, Rey Juan Carlos University, 28922 Madrid, Spain; ana.lopez@urjc.es (A.L.-d.-A.); valentin.hernandez@urjc.es (V.H.-B.); 4 Respiratory Department, Ramón y Cajal Hospital and Instituto Ramón y Cajal de Investigación Sanitaria (IRYCIS), Medicine Department, Universidad de Alcalá, CIBER de Enfermedades Respiratorias (CIBERES), 28038 Madrid, Spain; djimenez.hrc@gmail.com; 5 Department of Internal Medicine, Hospital Universitari Germans Trias i Pujol, CIBER de Enfermedades Respiratorias (CIBERES), 08916 Barcelona, Spain; mmonreal.germanstrias@gencat.cat

**Keywords:** pulmonary embolism, trends, incidence, in-hospital mortality, COPD, diabetes

## Abstract

(1) Background: The aims of this study were to examine trends in the incidence, clinical characteristics, and in-hospital outcomes of patients hospitalized with pulmonary embolism in Spain and to identify factors associated with in-hospital mortality (IHM). (2) Methods: We included all patients who were hospitalized for pulmonary embolism between 2001 and 2018. Data were collected from the Spanish National Hospital Discharge Database. (3) Results: We identified 241,821 hospitalizations for pulmonary embolism during the study period. The incidence of pulmonary embolism increased from 20.49 cases per 100,000 inhabitants in the period 2001–2002 to 35.9 cases in the period 2017–2018 (*p* < 0.001). After controlling for possible confounders, there was a significant increase in the incidence over the study period (adjusted incidence rate ratio 1.53, 95% Confidence Interval I 1.51–1.56). The median length of hospital stay was 11 days in the period 2001–2002, decreasing to seven days in the period 2017–2018 (*p* < 0.001). For the total time period, the crude IHM rate was 9.51%. After multivariable adjustment, IHM decreased significantly over time. The IHM was significantly higher in women, in patients suffering from more comorbidities, and in those with a massive pulmonary embolism. (4) Conclusions: Our results revealed an increase in the incidence of pulmonary embolism hospitalizations from 2001 to 2018 in Spain, with older patients being the most affected.

## 1. Introduction

Pulmonary embolism (PE), a potentially life-threatening complication of venous thromboembolism (VTE), is a major health problem worldwide. It is associated with significant morbidity and mortality among affected patients [1,2]. In fact, it is the third leading cardiovascular cause of death after coronary artery disease and stroke [3].

Longitudinal data have revealed an increasing tendency in annual PE incidence rates over time [4,5,6,7], although data from recent years are lacking. This finding could be due to an increasing number of patients with severe comorbidities and a higher risk of developing this complication, including cancer, the use of more accurate diagnostic imaging tests, and a lower threshold for disease suspicion [8]. By contrast, time trend analyses have pointed out that mortality of acute PE may be decreasing [4,5,6,7,8,9,10]. Possible explanations include increased or earlier diagnosis, use of more effective therapies, and better adherence to guidelines [10,11]. Nevertheless, in the modern era, there has been a tendency towards overdiagnosis of PE, since the introduction of computed tomographic pulmonary angiography (CTPA) to establish the diagnosis, which could be responsible for a decrease in case fatality [12]. In this scenario, increased diagnosis of trivial, or perhaps non-existent, PE may artificially reduce case-fatality rates. Unfortunately, previous studies have not elucidated this critical issue [13].

Although PE prognosis is improving, it has been demonstrated that annual PE incidence and PE-related mortality rates increase exponentially with age [2]. Given that the population is ageing rapidly, it is essential to evaluate trends in patients hospitalized for PE, stratified by age [14].

Real-world data are important for understanding the management of PE patients, particularly because a significant percentage of them have at least one exclusion criterion preventing their recruitment into randomized clinical trials [15]. However, existing data from clinical practice only provide limited information regarding some aspects such as PE severity. Additionally, management strategies may be changing over time, and recent national data on this topic are lacking. Thus, we hypothesized that the incidence and outcomes of patients hospitalized with PE may have also evolved.

The aims of the present study were (a) to examine trends in the incidence, clinical characteristics, and in-hospital outcomes of patients hospitalized with PE from 2001 to 2018 in Spain and (b) to identify factors associated with in-hospital mortality (IHM) among patients with PE in Spain over an 18-year study period.

## 2. Materials and Methods

### 2.1. Design, Setting, and Participants

We conducted an observational retrospective epidemiological study using the Spanish National Hospital Discharge Database (SNHDD) between 01 January 2001 and 31 December 2018. The SNHDD contains de-identified clinical and resource utilization data of over 95% of hospital discharges per year in Spain. Details of the database are described elsewhere [16].

The International Classification of Diseases, Ninth Revision, Clinical Modification (ICD-9-CM) was used for coding from 2001 to 2015, and for 2016 and 2017, the SNHDD used the 10th Revision (ICD-10). Hospital outcome variables such as the length of hospital stay (LOHS), costs, and IHM were collected by the SNHDD.

For study purposes, we used the definition of PE described by Smith et al. [17] (Supplementary Table S1).

### 2.2. Study Variables

To assess the comorbidity burden, all conditions included in the Charlson comorbidity index (CCI) coded in any diagnostic position in the discharge report were identified using the ICD-9-CM and ICD-10 codes, as described by Quan et al. [18]. We also showed the specific prevalence of the following conditions: valvular heart disease, hypertension, obesity, coagulopathy, and non-septic shock. Massive PE was defined as described by Smith et al. [17] and included subjects with documentation of mechanical ventilation, and/or vasopressors, and/or non-septic shock. The codes used to create these variables are shown in Supplementary Table S2.

The SNHDD includes a variable with the diagnosis-related group (DRG) categorized as medical/surgical/other. This was used to identify patients who underwent any type of surgical procedure during their hospital admission [16].

We specifically identified the following procedures: invasive mechanical ventilation (IMV), non-invasive mechanical ventilation (NIMV), thrombolytic therapy, inferior vena cava (IVC) filter placement, and vasopressor medication. The ICD-9-CM and ICD-10 codes used for these procedures are shown in Supplementary Table S2.

Costs were calculated using DRGs for the disease [19].

IHM was defined by the proportion of patients who died during admission for each time period analyzed.

### 2.3. Statistical Methods

We considered nine time periods and each period included two consecutive years. We estimated the incidence rates of PE admission calculated per 100,000 inhabitants to assess time trends by dividing the number of cases per year, sex, and age group by the corresponding number of people in that population group, according to the data from the Spanish National Institute of Statistics, as reported on 31 December of each year [20]. Trends in incidence were assessed using Poisson regression models adjusted by sex and age when required. The results are shown as the incidence rate ratio (IRR) with 95% confidence intervals (CIs).

A descriptive statistical analysis was performed for all continuous variables and categories. Variables are expressed as proportions, as means with standard deviations, and as medians with interquartile ranges. A bivariable analysis according to year was performed using the χ2 test for linear trends (proportions), ANOVA (means). or Kruskal–Wallis test (medians), as appropriate.

A multivariable logistic regression model was constructed to identify predictors of IHM among patients with PE providing odds ratios (ORs) with 95% CIs.

Stata version 14 (Stata, College Station, TX, USA) was used for data analysis.

### 2.4. Sensitivity Analysis

In our investigation, we used the definition of PE described by Smith et al. [16] using ICD-9-CM codes and the equivalent codes for ICD10 [16]. This definition excluded patients with acute cor pulmonale (ACP) because, the authors suggested that acute cor pulmonale was unlikely to be related solely to acute PE [16]. However, ACP is a clinical condition by which PE patients may ultimately die. For this reason, we analyzed hospitalization with ACP in Spain from 2001 to 2018 and the effect on our results if patients suffering ACP were included in the definition of PE. The codes used to identify ACP were 415.0. (ICD-9-CM) and I26.02 and I26.09 (ICD10).

### 2.5. Ethical Aspects

According to the Spanish legislation, because we used the SNHDD, a de-identified retrospective public access database which is provided freely to all investigators by the Spanish Ministry of Health, it was not necessary to obtain approval by an ethics committee or informed consent by the patients. The research was conducted according to the principles of the World Medical Association Declaration of Helsinki.

## 3. Results

### 3.1. Time Trends in Pulmonary Embolism Hospitalizations

The total number of hospital admissions in Spain with a PE diagnosis between 2001 and 2018 was 241,821 (92.8% with a primary diagnosis and 7.2% with a secondary diagnosis).

We found that the incidence of PE coding increased significantly from 20.49 cases per 100,000 inhabitants in the period 2001–2002 to 35.9 cases in the period 2017–2018 (*p* < 0.001). Over the entire period, the incidence of PE was higher in women than in men (31.72 per 100,000 inhabitants vs. 27.79 per 100,000 inhabitants, respectively, *p* < 0.001). Over time, the incidence of PE increased significantly in men and in women (18.97 and 21.96 per 100,000 inhabitants in the period 2001–02 vs. 34.2 and 37.75 per 100,000 inhabitants in the period 2017–2018, respectively, both *p* < 0.001). In every single time period, incidence rates were always higher among women.

Incidence rates were highest in older patients (146.44 per 100,000 inhabitants for those aged 70 years or older), with a value over four-fold higher than that in the 50–69 year group (33.91 per 100,000 inhabitants). There was a significant increase over time in the incidence in all age groups analyzed (all *p* < 0.001) (Table 1).

### 3.2. Distribution of Study Covariates among Patients Hospitalized with Pulmonary Embolism

Table 2 shows the age, CCI values, and clinical characteristics of the patients included in the study.

Age increased significantly over time (69.99 ± 15.43 years in the period 2001–2002 vs. 70.62 ± 15.78 years in the period 2017–2018, *p* < 0.001). We detected a significant increase in the number of comorbidities according to the mean CCI value over time (0.77 ± 0.86 comorbidities in the period 2001–02 vs. 0.99 ± 1.01 comorbidities in the period 2017–2018, *p* < 0.001). Over the entire time period, the most frequent associated comorbidities for patients hospitalized for PE were hypertension (38.49%), COPD (21.34%), cancer (16.37%), and diabetes (15.71%). The prevalence of cancer rose 1.57-fold, diabetes 1.4-fold, valvular heart disease 1.55-fold, and hypertension 1.34-fold. In addition to these conditions, and as seen in Table 2, the frequency of most conditions analyzed increased over time (*p* < 0.001); however, over time, we found a small but significant reduction in acute myocardial infarction, cerebrovascular disease, COPD, peptic ulcer disease, and non-septic shock.

The proportion of patients with massive PE slightly increased from 2001–2002 to 2017–2018 (3.12% vs. 3.66%, *p* < 0.001).

The procedures and in-hospital outcomes of patients hospitalized with PE are shown in Table 3.

We observed a significant decrease in the percentage of patients who had undergone surgery, from 2.75% in the period 2001–2002 to 1.75% in the period 2017–2018.

The use of NMIV in patients with PE increased significantly from 0.37% in the period 2001–02 to 1.63% in 2017; however, the use of IMV remained unchanged.

We observed a significant over three-fold increase in the use of thrombolytic therapy over time (1.83% in the period 2001–2002 vs. 6.04% in the period 2017–2018, *p* < 0.001).

The median LOHS for PE admissions was 11 days in the period 2001–02, decreasing to seven days in the period 2017–2018 (*p* < 0.001). In addition, the mean cost per patient increased from 3650 Euros in the period 2001–2002 to 4642 Euros in the period 2017–2018. For the total time period, the crude IHM rate was 9.51%. Overall, the crude IHM rate decreased significantly (*p* < 0.001) over time from 12.34% in the period 2001–2002 to 7.32% in the period 2017–2018.

### 3.3. Evolution of IHM among Patients Hospitalized with Pulmonary Embolism

As shown in Supplementary Figure S1, the IHM rate decreased significantly in women and in men from 12.88% and 11.68% in the period 2001–2002 to 7.42% and 7.21%, respectively, *p* < 0.001. The IHM was significantly higher in women than in men in all time periods analyzed. The IHM was highest in the older age groups (≥70 years) in all the study periods and decreased in all age groups over time.

### 3.4. Multivariable Analysis for Incidence and IHM in Patients Hospitalized with Pulmonary Embolism from 2001 to 2018

After controlling for possible confounders using Poisson regression models, there was a significant increase in incidence from 2001–2002 to 2017–2018 with an adjusted IRR of 1.53 (95% CI 1.51–1.56).

As shown in Table 4, among patients with PE, after multivariable adjustment, the IHM decreased significantly over time. The probability of dying in the hospital in the period 2017–2018 was half (OR 0.48, 95% CI 0.45–0.51) as compared with the period 2001–2002.

The IHM was significantly higher in those suffering from more comorbidities (OR 3.89, 95% CI 3.69–4.1 for those with a CCI value >2) and with massive PE (OR 9.87, 95% CI 9.43–10.34). Female sex increased the probability of dying (OR 1.04, 95% CI 1.01–1.08).

### 3.5. Sensitivity Analysis

The results of the sensitivity analysis including ACP in the definition of PE are shown in Supplementary Table S3. Over the entire period, 6158 subjects were hospitalized with a code for ACP, which represented 2.48% of all the admission with PE (247,979). As can be seen in the table, the proportion of patients with PE who suffered ACP decreased significantly from the period 2001–2002 (4.78%) to the period 2017–2018 (1.51%), for the entire sample and also among men and women. The total incidence rates per 100,000 inhabitants for PE including ACP increased from 21.52 in 2001–2002 to 36.45 in 2017–2018 (*p* < 0.001). These rates were slightly higher than those found for PE without ACP (20.49 and 35.9, respectively, Table 1) and with an identical time trend. Regarding IHM, those cases with ACP had figures of 11.01% in the period 2001–2002 and decreased to 8.01% in the period 2017–2018 (*p* = 0.002), the equivalent figures for patients with PE not including ACP were 12.34% and 7.32%, respectively.

## 4. Discussion

To our knowledge, this is the longest longitudinal analysis to date that examines the recent trends in hospital admissions for PE in Spain. We found a continued increase in the incidence of PE hospitalizations from 2001–2002 to 2017–2018. In parallel, the IHM decreased in the same period, being higher in women, in patients suffering from more comorbidities, and in those with massive PE.

Similar trends to ours in incidence and mortality have been described in previous studies [4,9,14,16,21,22,23]. Some authors have hypothesized that these findings reflect a movement towards hospital admission for less severe PE [17]. However, we found that the proportion of patients with massive PE slightly increased over time, which contradicts this hypothesis. In addition, the proportion of patients needing NIMV increased significantly from 2001–2002 to 2016–2017, and the use of IMV remained unchanged. It has also been suggested that increased admission rates may be related to the increased sensitivity and frequency of CTPA use [12]. In this way, Feng et al. [24] demonstrated that CTPA use in emergency departments in the USA levelled off after 2007, and our results showed that PE hospitalizations have continued to increase subsequently. We believe that the increased incidence of hospital admissions for PE over time may partially be due to other factors observed in our study, such as the progressive ageing of the population and the increase in some comorbidities that are known risk factors for this disease, such as cancer.

Our data revealed that the incidence of hospital admissions for PE was higher in women than in men, although the incidence increased significantly in both men and women. Raptis et al. [25] also found a female predominance in PE incidence, which was mainly attributed to age groups above 70 years. One explanation could be the existence of differences in life expectancy between the sexes [25]. Furthermore, differences in thrombotic and fibrinolytic activity between men and women may influence the sex-related discrepancies detected in PE incidence in older age individuals [26]. In addition, female sex increased the probability of dying in our study. However, both the point estimate of the risk (1.04) and the range (1.01–1.08) raise a question regarding the significance of the association and whether or not it is due solely to chance.

It has been reported that the incidence of hospitalizations for PE grows exponentially with age [21,27]. We found that the incidence of PE hospitalizations was also highest in patients older than 70 years as compared with younger patients. Regarding IHM, it significantly improved across all ages, with elderly patients also suffering from higher mortality rates in our study. Due to the progressive ageing of the population worldwide, it is expected that the overall medical, societal, and economic burden related to hospital admissions and deaths due to PE will continue to grow in the future. Unfortunately, elderly patients are typically excluded from randomized clinical trials on PE, therefore, there is still a significant knowledge gap regarding this population [14].

Our results document a significant increase in the number of comorbidities over time, consistent with findings of previous studies [14,28]. Among the comorbidities that significantly increased over time were hypertension, diabetes, obesity, cancer, and chronic kidney disease, some of which are known risk factors for PE [29,30,31]. Despite an increasing burden of comorbidities for PE patients, the LOHS improved over time in our study, as other authors have described [14,17]. Although some health systems have provided an incentive for shortening the LOHS, some factors that may contribute to these trends are the more frequent use of the option of early discharge for selected low-risk patients and the advances in PE treatment, including the availability of low-molecular-weight heparin [9] and direct oral anticoagulants [14], which are related to a decreased LOHS [32,33,34].

Little is known about the current use of reperfusion therapy in PE patients in the real world. We observed a significant increase (over three-fold) in the use of thrombolysis over time. More frequent use of thrombolytic treatments may have contributed to improved IHM rates in patients with high-risk PE. Other authors have also described an increasing trend in the use of these agents and have related it to growing attention in the literature and the inclusion of thrombolysis in clinical guidelines as a standard reperfusion therapy for patients with stable haemodynamic PE in the absence of contraindications [7,35]. Patient characteristics associated with the administration of this therapy include younger age, male sex, white race, residence in a higher-income zone, and treatment in a large academic hospital [35]. However, while disparities in receiving tPA may mediate differences in outcomes for patients with PE, all of those factors are also associated with increased morbidity and mortality from other cardiopulmonary diseases and may actually act as confounders in this association between tPA and IHM rates.

Until recently, IVC filters were supposed to be efficacious, and in fact, they were employed for many patients to prevent subsequent PE. Nevertheless, some recent studies have cast doubt on the efficacy of these devices [36]. In this sense, Bikdeli et al. [23] recently recorded a decline in the number and rates of IVC filter utilization in the USA, since 2010. They suggested that the causes of these trends were likely multifactorial, including concerns about the safety and efficacy of these devices, educational initiatives of scientific societies, or changes in reimbursement policies. Stein et al. [37] also reported that the proportion of patients who receive an IVC filter was decreasing, both in stable and unstable PE patients. In the current study, we did not detect significant changes in its use in Spain over time.

We observed a continuous decrease in IHM from 2001 to 2018, a finding consistent with the decreasing trend reported by Barco et al. when they analyzed vital registration data from the WHO Mortality Database (2000–2015) [8]. Among potential explanations are the temporal changes in pharmacological and interventional treatments for PE [9] and more effective referral to high-volume centers [38].

Our findings demonstrated that hospitalization costs increased over the study period, despite a decreasing LOHS and mortality. Increasing comorbidities could partially justify the increased costs. It could also be the reflection of an older, sicker population, given that the mean age of patients has increased significantly over time. Similar trends have been noted by other authors in the USA [14,17].

The strengths of this investigation include the large number of patients evaluated, which permits further stratification according to age and sex, and the large number of years in which trends for PE hospitalizations are shown. Nevertheless, our study has several limitations, some of which are inherent to the study design and the use of administrative data. First, it was based on ICD discharge codes rather than clinical criteria, which could be subject to underreporting or miscoding [39]. Nevertheless, the sensitivity and specificity of the diagnosis codes for a principal discharge diagnosis of PE are high as compared with objectively documented diseases based on medical chart review criteria [40]. We used the definition of PE suggested by Smith et al. [16] which excluded ACP codes and this decision may affect our results. However, when we conducted a sensitivity analysis including ACP in the codes for PE, we found that the importance of this condition in the incidence, time trends, and IHM is very small and in our opinion does not affect the main conclusion of our investigation.

Second, detailed clinical data, such as laboratory parameters or pharmacological treatments, were not available. Third, we acknowledge that information on the cause of death could not be obtained from the Spanish nationwide inpatient sample, so the mortality assessment was limited to all-cause hospital mortality. However, previous studies have demonstrated that IHM is mostly related to acute PE episodes, in contrast to other causes of death, such as comorbidities, which affect mortality over the long term [7]. Despite these limitations, our analysis helps address the current gap in the literature regarding the epidemiology of hospital admissions for PE, in Spain.

## 5. Conclusions

In conclusion, real-world data using a large national database demonstrate an increase in the incidence of PE hospitalizations from 2001 to 2018 in Spain, both overall and according to sex and age group, with older patients being the most affected. Despite improvements in the LOHS and IHM, hospitalization costs increased.

This disease remains a clinically significant cause of mortality for which both increased awareness and preventive actions are needed.

## Figures and Tables

**Table 1:** Distribution according to diagnosis position and age-sex specific incidence rates for pulmonary embolism (PE) in Spain from 2001 to 2018.

	Time Periods									
	2001–02	2003–04	2005–06	2007–08	2009–10	2011–12	2013–14	2015–16	2017–18	Total
Admissions with PE as Primary Diagnosis, *n* (%)	15,899 (94.52)	18,464 (94.54)	19,636 (93.3)	22,415 (89.98)	26,464 (90.52)	28,693 (91.76)	30,232 (93.01)	30,875 (93.51)	31,740 (94.78)	224,418 (92.8)
Admissions with PE as Secondary Diagnosis, *n* (%)	922 (5.48)	1066 (5.46)	1411 (6.7)	2496 (10.02)	2771 (9.48)	2576 (8.24)	2272 (6.99)	2142 (6.49)	1747 (5.22)	17,403 (7.2)
*n* Male, (Rate per 100,000) *	7634 (18.97)	8863 (21.19)	9690 (22.31)	11,472 (25.43)	13,592 (29.58)	14,085 (30.53)	14,849 (32.44)	15,269 (33.48)	15,645 (34.2)	111,099 (27.79)
Females, *n* (Rate per 100,000) *	9187 (21.96)	10,667 (24.68)	11,357 (25.48)	13,439 (29.15)	15,643 (33.29)	17,184 (36.27)	17,655 (37.35)	17,748 (37.56)	17,842 (37.55)	130,722 (31.72)
<50 Years, *n* (Rate per 100,000) *	1964 (3.56)	2215 (3.88)	2434 (4.13)	3010 (4.95)	3605 (5.89)	3615 (5.98)	3785 (6.44)	3639 (6.35)	3735 (6.63)	28,002 (5.33)
50–69 Years, *n* (Rate per 100,000) *	4451 (25.85)	4811 (27.23)	5036 (27.6)	5818 (30.45)	7029 (34.74)	7885 (37.16)	8500 (38.5)	8746 (38.17)	9608 (40.35)	61,884 (33.91)
≥70 Years, *n* (Rate per 100,000) *	10,406 (106.72)	12,504 (120.62)	13,577 (124.71)	16,083 (141.85)	18,601 (161.31)	19,769 (166.77)	20,219 (165.5)	20,632 (162.66)	20,144 (153.47)	151,935 (146.44)
Total, *n* (Rate per 100,000) *	16,821 (20.49)	19,530 (22.96)	21,047 (23.91)	24,911 (27.31)	29,235 (31.46)	31,269 (33.44)	32,504 (34.93)	33,017 (35.55)	33,487 (35.9)	241,821 (29.78)

Incidence rates calculated by dividing the number of cases per year age group with the corresponding number of persons in that population group according to the National Institute of Statistics (INE) reported on December 31 each year. * Significant time trend (*p* < 0.001) estimated using Poisson regression models adjusted by age and sex as required.

**Table 2:** Age, Charlson comorbidity index (CCI), and clinical characteristics of patients hospitalized with pulmonary embolism in Spain from 2001 to 2018.

	2001–02	2003–04	2005–06	2007–08	2009–10	2011–12	2013–14	2015–16	2017–18	Total	*p*-Value
Age, mean (SD)	69.99 (15.43)	70.5 (15.52)	70.63 (15.65)	70.64 (15.89)	70.72 (15.96)	71.05 (15.79)	70.91 (15.90)	71.24 (15.71)	70.62 (15.78)	70.76 (15.77)	<0.001
CCI, mean (SD)	0.77 (0.86)	0.85 (0.91)	0.88 (0.92)	0.91(0.93)	0.96 (0.94)	1 (0.96)	1.02 (0.98)	0.98 (0.98)	0.99 (1.01)	0.95 (0.96)	<0.001
CCI = 0, *n* (%)	7673 (45.62)	8205 (42.01)	8544 (40.59)	9746 (39.12)	1,0754 (36.78)	1,1062 (35.38)	11,329 (34.85)	12,230 (37.04)	12,567 (37.53)	92,110 (38.09)	<0.001
CCI 1–2, *n* (%)	8453 (50.25)	10,290 (52.69)	11,327 (53.82)	13,660 (54.84)	16,594 (56.76)	17,887 (57.2)	18,631 (57.32)	18,302 (55.43)	18,168 (54.25)	133,312 (55.13)	
CCI >2, *n* (%)	695 (4.13)	1035 (5.3)	1176 (5.59)	1505 (6.04)	1887 (6.45)	2320 (7.42)	2544 (7.83)	2485 (7.53)	2752 (8.22)	16,399 (6.78)	
AMI, *n* (%)	465 (2.76)	596 (3.05)	604 (2.87)	719 (2.89)	668 (2.28)	619 (1.98)	596 (1.83)	653 (1.98)	898 (2.68)	5818 (2.41)	<0.001
CHF, *n* (%)	1479 (8.79)	1658 (8.49)	1982 (9.42)	2415 (9.69)	2974 (10.17)	3334 (10.66)	3396 (10.45)	3580 (10.84)	3699 (11.05)	24,517 (10.14)	<0.001
PVD, *n* (%)	521 (3.1)	731 (3.74)	783 (3.72)	869 (3.49)	1032 (3.53)	1161 (3.71)	1229 (3.78)	1166 (3.53)	1225 (3.66)	8717 (3.6)	0.008
CVD, *n* (%)	738 (4.39)	950 (4.86)	885 (4.2)	1085 (4.36)	1318 (4.51)	1341 (4.29)	1388 (4.27)	1267 (3.84)	1299 (3.88)	10,271 (4.25)	<0.001
Dementia, *n* (%)	637 (3.79)	847 (4.34)	941 (4.47)	1105 (4.44)	1371 (4.69)	1515 (4.85)	1399 (4.3)	1695 (5.13)	1944 (5.81)	11,454 (4.74)	<0.001
COPD, *n* (%)	3309 (19.67)	4144 (21.22)	4448 (21.13)	5318 (21.35)	6603 (22.59)	7291 (23.32)	7733 (23.79)	7030 (21.29)	5732 (17.12)	51,608 (21.34)	<0.001
Rheumatoid Disease, *n* (%)	298 (1.77)	410 (2.1)	466 (2.21)	601 (2.41)	760 (2.6)	799 (2.56)	971 (2.99)	903 (2.73)	861 (2.57)	6069 (2.51)	<0.001
PUD, *n* (%)	219 (1.3)	178 (0.91)	170 (0.81)	182 (0.73)	158 (0.54)	179 (0.57)	139 (0.43)	132 (0.4)	137 (0.41)	1494 (0.62)	<0.001
Liver Disease, *n* (%)	413 (2.46)	649 (3.32)	770 (3.66)	1000 (4.01)	1362 (4.66)	1556 (4.98)	1699 (5.23)	1727 (5.23)	1844 (5.51)	11,020 (4.56)	<0.001
Diabetes, *n* (%)	2093 (12.44)	2770 (14.18)	3115 (14.8)	3726 (14.96)	4468 (15.28)	5110 (16.34)	5487 (16.88)	5413 (16.39)	5819 (17.38)	38,001 (15.71)	<0.001
HP/PAPL, *n* (%)	72 (0.43)	96 (0.49)	121(0.57)	147 (0.59)	165 (0.56)	175 (0.56)	207 (0.64)	161 (0.49)	181 (0.54)	1325 (0.55)	0.086
Renal Disease, *n* (%)	704 (4.19)	1024 (5.24)	1174 (5.58)	1565 (6.28)	2100 (7.18)	2479 (7.93)	2974 (9.15)	2999 (9.08)	3338 (9.97)	18,357 (7.59)	<0.001
Cancer, *n* (%)	1955 (11.62)	2542 (13.02)	3004 (14.27)	3922 (15.74)	4964 (16.98)	5523 (17.66)	5859 (18.03)	5695 (17.25)	6116 (18.26)	39,580 (16.37)	<0.001
AIDS, *n* (%)	31 (0.18)	34 (0.17)	34 (0.16)	43 (0.17)	43 (0.15)	49 (0.16)	56 (0.17)	57 (0.17)	71 (0.21)	418 (0.17)	0.772
Valvula Heart Disease, *n* (%)	689 (4.1)	867 (4.44)	962 (4.57)	1186 (4.76)	1651 (5.65)	1741 (5.57)	1874 (5.77)	1901(5.76)	2132 (6.37)	13,003 (5.38)	<0.001
Hypertension, *n* (%)	5114 (30.4)	6858 (35.12)	7820 (37.15)	9484 (38.07)	11,441 (39.13)	12,380 (39.59)	13,003 (40)	13,343 (40.41)	13,641 (40.74)	93,084 (38.49)	<0.001
Obesity, *n (*%)	1308 (7.78)	1774 (9.08)	2040 (9.69)	2608 (10.47)	3139 (10.74)	3560 (11.39)	3978 (12.24)	3986 (12.07)	4401 (13.14)	26,794 (11.08)	<0.001
Coagulopathy, *n* (%)	174 (1.03)	225 (1.15)	185 (0.88)	220 (0.88)	201 (0.69)	218 (0.7)	228 (0.7)	427 (1.29)	609 (1.82)	2487 (1.03)	<0.001
Nonseptic Shock, *n* (%)	288 (1.71)	365 (1.87)	290 (1.38)	316 (1.27)	379 (1.3)	419 (1.34)	414 (1.27)	341 (1.03)	349 (1.04)	3161 (1.31)	<0.001
Massive PE, *n* (%)	524 (3.12)	708 (3.63)	681 (3.24)	867 (3.48)	1061 (3.63)	1312 (4.2)	1423 (4.38)	1294 (3.92)	1225 (3.66)	9095 (3.76)	<0.001

CCI, Charlson comorbidity index; AMI, acute myocardial infarction; CHF, congestive heart failure; PVD, peripheral vascular disease; CVD, cerebrovascular disease; COPD, chronic obstructive pulmonary disease; PUD, peptic ulcer disease; T2DM, type 2 diabetes mellitus; HP/PAPL, hemiplegia/paraplegia; AIDS, acquired immune deficiency syndrome; PE, pulmonary embolism. *p*-Value for time trend using χ2 test for linear trend (proportions) or ANOVA (means).

**Table 3:** Procedures and in-hospital outcomes for patients hospitalized with pulmonary embolism in Spain from 2001 to 2018.

	2001–02	2003–04	2005–06	2007–08	2009–10	2011–12	2013–14	2015–16	2017–18	Total	*p*-Value
Undergone Surgery, *n* (%)	463 (2.75)	466 (2.39)	376 (1.79)	306 (1.23)	339 (1.16)	416 (1.33)	442 (1.36)	576 (1.74)	587 (1.75)	3971 (1.64)	0.006
IMV, *n* (%)	273 (1.62)	390 (2)	338 (1.61)	416 (1.67)	428 (1.46)	463 (1.48)	473 (1.46)	462 (1.4)	522 (1.56)	3765 (1.56)	0.054
NIMV, *n* (%)	62 (0.37)	131 (0.67)	162 (0.77)	252 (1.01)	410 (1.4)	623 (1.99)	731 (2.25)	663 (2.01)	547 (1.63)	3581 (1.48)	0.021
Thrombolytic Therapy, *n* (%)	307 (1.83)	453 (2.32)	472 (2.24)	568 (2.28)	749 (2.56)	959 (3.07)	1055 (3.25)	1297 (3.93)	2024 (6.04)	7884 (3.26)	<0.001
IVC Filter Placement, *n* (%)	237 (1.41)	220 (1.13)	225 (1.07)	246 (0.99)	294 (1.01)	344 (1.1)	335 (1.03)	302 (0.91)	316 (0.94)	2519 (1.04)	0.071
Vasopressors Medication, *n* (%)	0 (0)	0 (0)	19 (0.09)	93 (0.37)	88 (0.3)	108 (0.35)	157 (0.48)	108 (0.33)	106 (0.32)	679 (0.28)	<0.101
LOHS, Median (IQR)	11 (8)	11 (8)	10 (7)	10 (8)	9 (7)	8 (6)	8 (6)	8 (6)	7 (6)	9 (7)	<0.001
Costs, Mean in Euros (SD)	3650.78 (27.07)	4313.87 (602.91)	4265.07 (113)	4363.74 (558.59)	4085.86 (890.32)	4040.28 (428.1)	4330.55 (257.52)	4624.14 (260.29)	4641.69 (785.44)	4300.85 (570.77)	<0.001
IHM, *n* (%)	2075 (12.34)	2416 (12.37)	2497 (11.86)	2667 (10.71)	2886 (9.87)	2834 (9.06)	2647 (8.14)	2529 (7.66)	2451 (7.32)	23002 (9.51)	<0.001

IMV, invasive mechanical ventilation; NIMV, non-invasive mechanical ventilation; IVC, inferior vena cava; LOHS, length of hospital stay; IHM, in-hospital mortality. *p*-Value for time trend using χ2 test for linear trend (proportions), ANOVA (means), or Kruskall–Wallis test (medians).

**Table 4:** Multivariate analysis for incidence and in-hospital mortality for patients hospitalized with pulmonary embolism (PE) in Spain from 2001 to 2018.

		Incidence (IRR) ^a^	In-Hospital Mortality (OR) ^b^
Age (years)	<50 Years	1	1
	50–69 Years	6.29 (6.2–6.38)	1.74 (1.61–1.87)
	≥70 Years	27.39 (27.04–27.74)	2.9 (2.71–3.11)
Sex	Men	1	1
	Female	1.07 (1.07–1.08)	1.04 (1.01–1.08)
Charlson	0	1	1
	1–2	1.45 (1.44–1.46)	2.24 (2.16–2.32)
	>2	0.18 (0.18–0.18)	3.89 (3.69–4.1)
Year	2001–02	1	1
	2003–04	1.12 (1.1–1.14)	0.94 (0.88–1)
	2005–06	1.16 (1.13–1.18)	0.89 (0.84–0.95)
	2007–08	1.31 (1.29–1.34)	0.76 (0.72–0.82)
	2009–10	1.5 (1.47–1.53)	0.68 (0.64–0.72)
	2011–12	1.56 (1.53–1.59)	0.59 (0.55–0.62)
	2013–14	1.58 (1.55–1.61)	0.51 (0.48–0.54)
	2015–16	1.56 (1.53–1.59)	0.49 (0.46–0.52)
	2017–18	1.53 (1.51–1.56)	0.48 (0.45–0.51)
Massive PE		0.04 (0.01–0.06)	9.87 (9.43–10.34)

IRR, Incidence Rate ratio; OR Odds Ratio; ^a^ Calculated using multivariate Poisson regression for dependent variable “incidence of hospitalizations”. ^b^ Calculated using logistic regression for dependent variable “ in-hospital mortality”. The independent variables included in the models are those shown in the table.

## Supplementary Materials

The following are available online at https://www.mdpi.com/2077-0383/9/10/3221/s1, Table S1: ICD-9-CM codes and ICD-10 codes used to define pulmonary embolism for study purpose based on Smith et al., Table S2: ICD-9-CM and ICD-10 codes for the clinical diagnosis and procedures used in this investigation, Table S3: Sensitivity analysis including acute cor pulmonale (ACP) in the definition of pulmonary embolism (PE). Number of cases, proportions, incidences rates, and in-hospital mortality (IHM) according to sex.
