# Supplementary material for: Changing Trends in Hospital Admissions for Pulmonary Embolism in Spain from 2001 to 2018
Source: J Clin Med. 2020 Oct 7;9(10):3221. doi: 10.3390/jcm9103221 (PMC7599502; doi:10.3390/jcm9103221)
Supplement: Supplementary file 1 [file jcm-09-03221-s001.zip › jcm-930607-Figures.pdf]

Supplementary Figure 1. In-hospital mortality rates (%) according to sex and age groups for pulmonary embolism hospitalizations in Spain from 2001 to 2018.

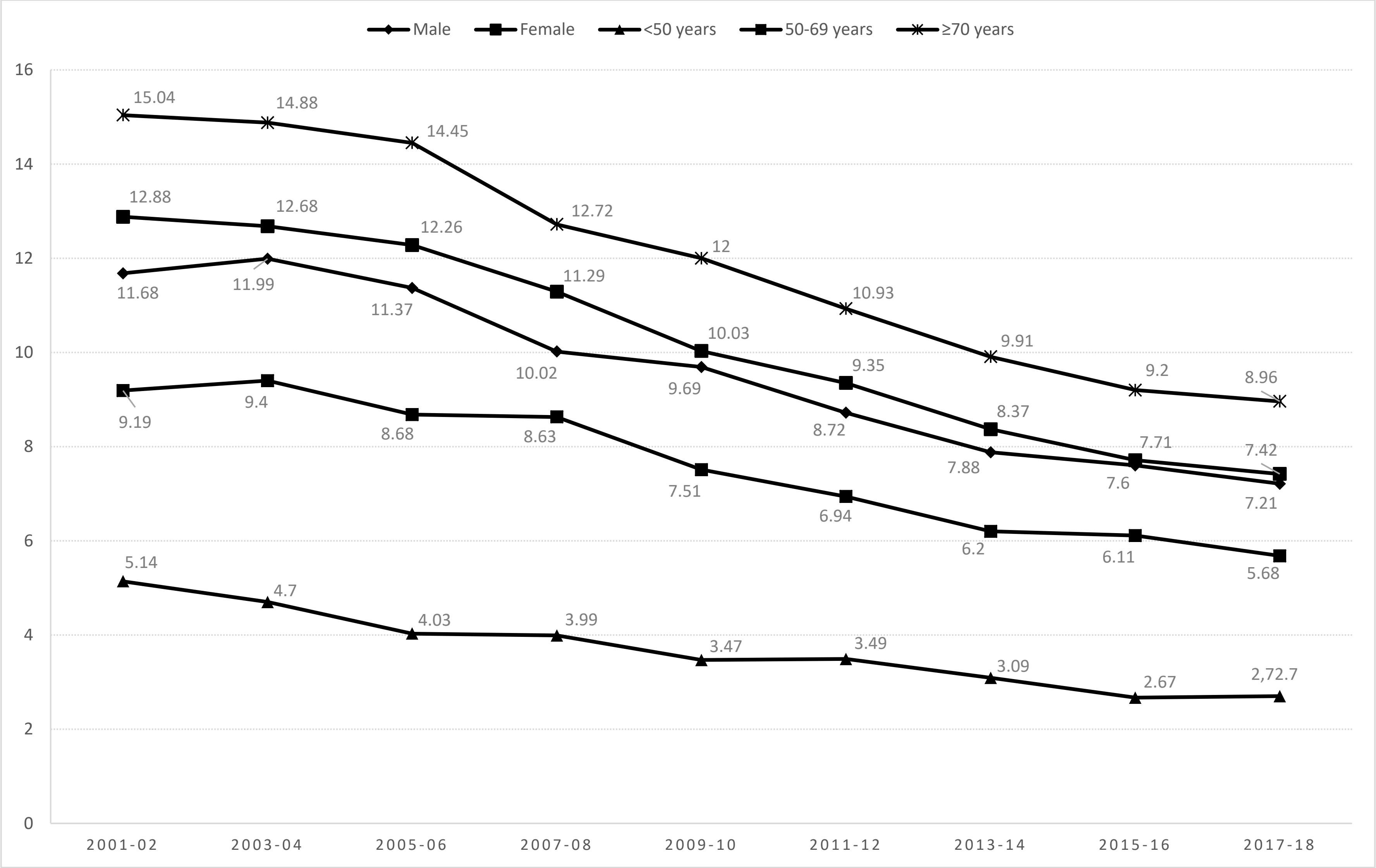

Significant time trend estimated using sex-age adjusted multivariable Poisson regression models for all categories.(p<0.001)
