# Supplementary material for: Changing Trends in Hospital Admissions for Pulmonary Embolism in Spain from 2001 to 2018
Source: J Clin Med. 2020 Oct 7;9(10):3221. doi: 10.3390/jcm9103221 (PMC7599502; doi:10.3390/jcm9103221)
Supplement: Supplementary file 1 [file jcm-09-03221-s001.zip › jcm-930607-Supplementary.docx]

**Supplementary Table 1:** ICD-9-CM codes and ICD-10 codes used to define pulmonary embolism for study purpose based on Smith et al^#^.

| **ICD-9-CM** | **ICD-10** |
| --- | --- |
| INCLUSION | |
| As primary diagnosis | |
| 415.1 Pulmonary embolism (PE)  415.13 Saddle embolus of pulmonary artery  415.19 Other PE | I26.92  I26.99 |
| Other principal diagnoses included if PE was listed as a secondary diagnosis | |
| 518.81 Acute respiratory failure  518.82 Other pulmonary insufficiency  518.84 Acute and chronic respiratory failure  799.1 Respiratory arrest  453.4x Acute DVT of lower extremities  453.8 Acute DVT of other specified veins  453.82 Acute DVT of upper extremities  453.83 Acute DVT of upper extremities NOS  453.9 Other DVT of unspecified site | J96.00, J96.90  J80, R06.03  J96.20  R09.2  I82.409, I82.4Y9, I82.419, I82.429, I82.439. I82.4Z9, I82.449, I82.499  I82.629,  I82.609  I82.91 |
| EXCLUSION |  |
| 415.0 Acute cor pulmonale  415.11 Iatrogenic PE and infarction  415.12 Septic PE  416.xx Chronic pulmonary heart disease  634.6, 639.6, 673.xxPEComplicating abortion* mola  V12.55 Personal history of PE | I26.09  I26.90; I26.99  I26.90  I27.0, I27.1, I27.82, I27. I27.21, I27.22, I27.23, I27.24, I27.29, I27.89  O03.7, O03.2, O03.7, O08.2, O88.019, O88.011, O88.012, O88.013, O88.02, O88.03  Z86.711 |

DVT: deep venous thrombosis * including molar pregnancies, pregnancy, or childbirth.

^#^Smith SB, Geske JB, Kathuria P, Cuttica M, Schimmel DR, Courtney DM, Waterer GW, Wunderink RG. Analysis of national trends in admissions for pulmonary embolism. *Chest.* **2016**;150(1):35-45.

**Supplementary Table 2:** ICD-9-CM and ICD-10 codes for the clinical diagnosis and procedures used in this investigation.

|  | **ICD-9-CM** | **ICD-10** |
| --- | --- | --- |
| Valvular heart disease | 394.x, 395.x, 396.x, 424.0, 424.1, 424.2, 424.3 | I05.X, I06.X, I07.X, I08.X, I34.X,I35.X, I36.X, I37.X |
| Hypertension | 401.X | I10, I16.6 |
| Obesity | 278.XX | E66.X |
| Coagulopathy | 286.XX | D68.XX |
| Nonseptic shock | 785.50, 785.51, 785.59 | R57.0, R57.1, R57.8, R57.9, |
|  |  |  |
| Invasive mechanical ventilation | 96.70, 96.71, 96.72 | 5A1945Z, 5A1955Z, 5A1935Z |
| Non-invasive mechanical ventilation | 93.90, 93.91 | 5A09357, 5A09457, 5A09557 |
| Thrombolytic therapy | 99.10 | 3E03317, 3E04317, 3E05317, 3E06317, 3E08317 |
| Inferior vena cava filter placement* | 38.7 | 02HV0DZ, 02HV3DZ, 02HV4DZ, 02LV0CZ, 02LV0DZ, 02LV0ZZ, 02LV3CZ, 02LV3DZ, 02LV3ZZ, 02LV4CZ,,02LV4DZ,,02LV4ZZ,,02VV0CZ , 02VV0DZ, 02VV0ZZ, 02VV3CZ, 02VV3DZ , 02VV3ZZ, 02VV4CZ, 02VV4DZ , 02VV4ZZ , 6H00DZ, 06H03DZ , 06H04DZ , 06L00CZ, 06L00DZ , 06L00ZZ , 06L03CZ, 06L03DZ , 06L03ZZ , 06L04CZ, 06L04DZ , 06L04ZZ , 06V00CZ, 06V00DZ ,06V00ZZ 06V03CZ, 06V03DZ, 06V03ZZ, 06V04CZ, 06V04DZ, 06V04ZZ |
| Vasopressors | 00.17 | 3E030XZ, 3E033XZ, 3E040XZ, 3E043XZ, 3E050XZ, 3E053XZ, 3E060XZ, 3E063XZ |

*The ICD-9-CM has a single code (38.7) for the procedure “Inferior vena cava filter placement”. However, the ICD-10 gives the option to provide detailed information on anatomical localization, approach and device so this makes many possible combinations for “Inferior vena cava filter placement”.

Supplementary Table 3. Sensitivity analysis including Acute Cor Pulmonale (ACP) in the definition of Pulmonary Embolism (PE). Number of cases, proportions, incidences rates and In-hospital Mortality (IHM) according to sex.

|  |  | 2001-02 | 2003-04 | 2005-06 | 2007-08 | 2009-10 | 2011-12 | 2013-14 | 2015-16 | 2017-18 | Total | p-value^#^ |
| --- | --- | --- | --- | --- | --- | --- | --- | --- | --- | --- | --- | --- |
| Men | Number of cases with ACP | 558 | 500 | 468 | 497 | 430 | 423 | 369 | 326 | 301 | 3872 |  |
|  | %* | 6.81 | 5.34 | 4.61 | 4.15 | 3.07 | 2.92 | 2.42 | 2.09 | 1.89 | 3.37 | <0.001 |
|  | Number of cases with PE including ACP | 8192 | 9363 | 10158 | 11969 | 14022 | 14508 | 15218 | 15595 | 15946 | 114971 |  |
|  | Incidence rate per 100.000 inhabitants | 20.36 | 22.39 | 23.39 | 26.53 | 30.52 | 31.45 | 33.25 | 34.19 | 34.86 | 28.76 | <0.001 |
| Women | Number of cases with ACP | 287 | 297 | 269 | 228 | 264 | 236 | 258 | 236 | 211 | 2286 |  |
|  | %* | 3.03 | 2.71 | 2.31 | 1.67 | 1.66 | 1.35 | 1.44 | 1.31 | 1.17 | 1.72 | <0.001 |
|  | Number of cases with PE including ACP | 9474 | 10964 | 11626 | 13667 | 15907 | 17420 | 17913 | 17984 | 18053 | 133008 |  |
|  | Incidence rate per 100.000 inhabitants | 22.65 | 25.37 | 26.08 | 29.64 | 33.85 | 36.77 | 37.90 | 38.06 | 37.99 | 32.27 | <0.001 |
| Both | Number of cases with ACP | 845 | 797 | 737 | 725 | 694 | 659 | 627 | 562 | 512 | 6158 |  |
|  | %* | 4.78 | 3.92 | 3.38 | 2.83 | 2.32 | 2.06 | 1.89 | 1.67 | 1.51 | 2.48 | <0.001 |
|  | Number of cases with PE including ACP | 17666 | 20327 | 21784 | 25636 | 29929 | 31928 | 33131 | 33579 | 33999 | 247979 |  |
|  | Incidence rate per 100.000 inhabitants | 21.52 | 23.90 | 24.75 | 28.10 | 32.21 | 34.14 | 35.60 | 36.16 | 36.45 | 30.54 | <0.001 |
| IHM | Number of cases with ACP who died | 93 | 89 | 82 | 91 | 73 | 65 | 52 | 46 | 41 | 632 |  |
|  | % | 11.01 | 11.17 | 11.13 | 12.55 | 10.52 | 9.86 | 8.29 | 8.19 | 8.01 | 10.26 | 0.002 |
|  | Number of cases with PE including ACP who died | 2075 | 2416 | 2497 | 2667 | 2886 | 2834 | 2647 | 2529 | 2451 | 23002 |  |
|  | % | 12.27 | 12.32 | 11.84 | 10.76 | 9.89 | 9.08 | 8.15 | 7.67 | 7.33 | 9.53 | <0.001 |

* Proportion calculated by dividing number of cases with ACP by the total number of cases with PE including ACP. Incidence rates calculated by dividing the number of cases per year age group with the corresponding number of persons in that population group according to the National Institute of Statistics (INE) reported at December 31 each year. ^#^Significant time trend (p<0.001) estimated using Poisson regression models adjusted by age and sex as required for incidence rates and χ2 test for linear trends. P trend for proportions.
